# Correction to “The Research of Transgenic Human Nucleus Pulposus Cell Transplantation in the Treatment of Lumbar Disc Degeneration”

**DOI:** 10.1002/kjm2.70152

**Published:** 2026-01-03

H.‐C. Wang, C.‐H. Jin, J. Kong J, et al., “The Research of Transgenic Human Nucleus Pulposus Cell Transplantation in the Treatment of Lumbar Disc Degeneration,” *Kaohsiung Journal of Medical Sciences* 35 (2019): 486–492, https://doi.org/10.1002/kjm2.12084.

In our published article “The research of transgenic human nucleus pulposus cell transplantation in the treatment of lumbar disc degeneration” (DOI: 10.1002/kjm2.12084), we identified partial image duplication between Figures 4C and 4I, as well as Figures 4D and 4E. Upon review of the original data, we determined that this duplication resulted from an operational error during figure preparation. Due to the large number of images with highly similar morphologies and repetitive patterns in the imaging process, some representative images were inadvertently duplicated. We sincerely apologize for this oversight and any inconvenience it may have caused.


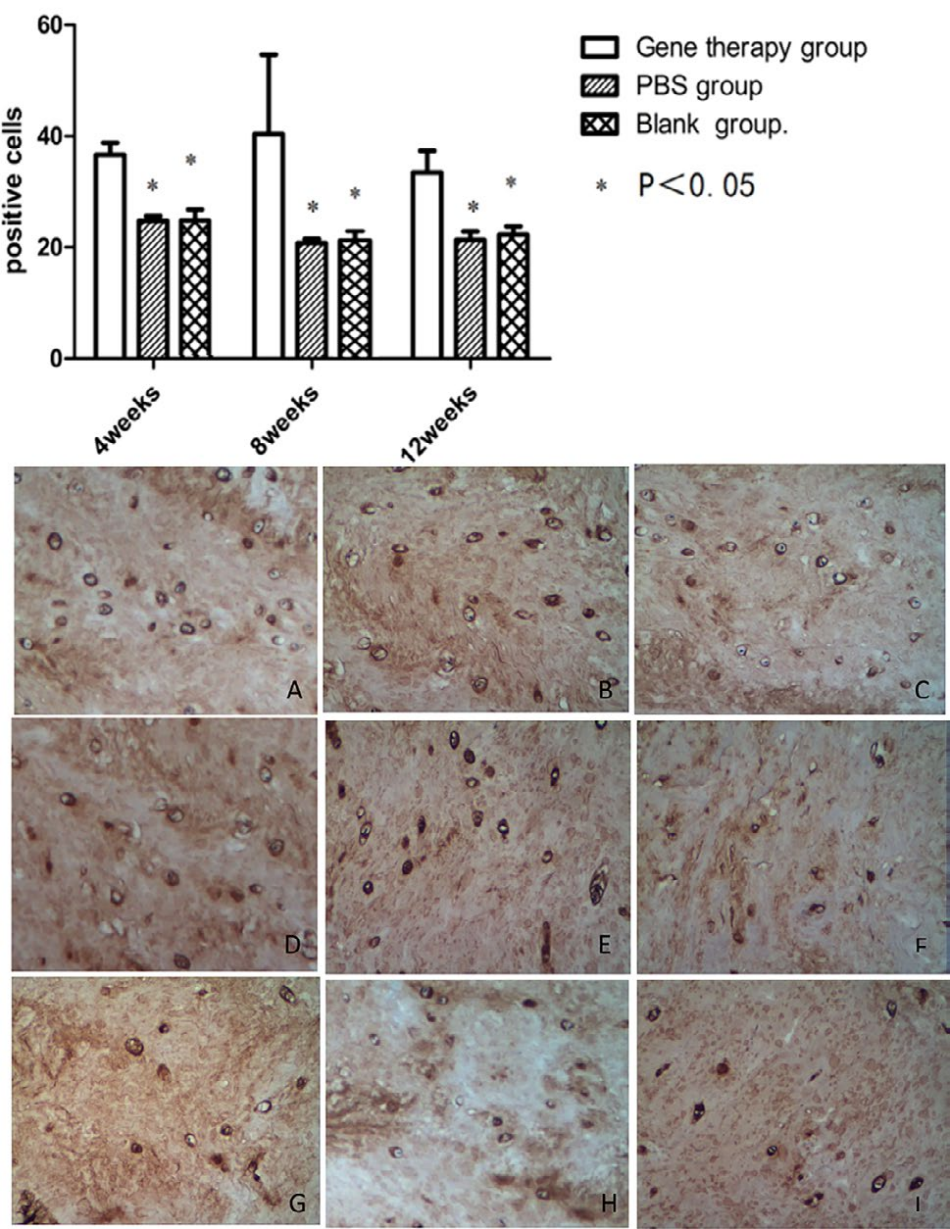


The authors confirmed that all results and conclusions of this article remain unchanged.

We apologize for this error.

